# 

**Published:** 1901-03

**Authors:** 


### BOOKS RECEIVED.

A Medico-Legal Manual. By William W. Keysor, Lecturer on Medical Jurisprudence in the Omaha Medical College and Judge of the District Court, Omaha, Nebraska. 12mo., pp. 316. Omaha: Burkley Printing Co. 1901. [Price, $2.00.]

A Text-Book on Practical Obstetrics. By Egbert H. Grandin, M. D., Gynecologist to the Columbus Hospital; Consulting Gynecologist to the French Hospital, etc. With the collaboration of George W. Jarman, M. D., Gynecologist to the Cancer Hospital. Third edition, revised and enlarged. Illustrated with 52 full-page photographic plates and 105 illustrations in the text. Pages xiv.—511. F. A. Davis Company, Philadelphia. 1901. [Price, cloth, $4.00, net; sheep, $4.75, net.]

Uterine Tumors; Their Pathology and Treatment. By W. Roger Williams, Fellow of the Royal College of Surgeons. 12mo., pp. 375. Illustrated. New York: William Wood & Company. 1901. [Price, $3.00 net.]

The Johns Hopkins Hospital Reports. Vol. VIII. Nos. 3–9. 1900.

Transactions of the Mississippi Valley Medical Association. Twenty-sixth annual session, held at Asheville, N. C., October 9, 10 and 11, 1900. Volume II. Henry E. Tuley, M. D., Secretary.

Fifteenth Annual Report of the State Board of Health and Vital Statistics of the Commonwealth of Pennsylvania. Vols. I. and II. 1899. Benjamin Lee, M. D., Secretary.

Obstetric and Gynecologic Nursing. By E. P. Davis, A. M., M. D., Professor of Obstetrics in Jefferson Medical College and Philadelphia Polyclinic. 12mo, 402 pages, fully illustrated. Philadelphia and London: W. B. Saunders & Co., 1901. [Price, $1 75, net.]

International Clinics. A Quarterly of Clinical Lectures and Especially Prepared Articles on Medicine, Neurology, Surgery, Therapeutics, Obstetrics, Pediatrics, Pathology, Dermatology, Diseases of the Eye, Ear, Nose and Throat, etc. Edited by Henry W. Cattell, A M., M. D., of Philadelphia, with the collaboration of John B. Murphy, M. D., of Chicago; Alexander D. Blackader, M. D., of Montreal; H. C. Wood, M. D., of Philadelphia; T. M. Rotch, M. D., of Boston; E Landolt, M. D., of Paris; Thomas G. Morton, M. D., and Charles H. Reed, M. D., of Philadelphia; J. W. Ballantyne, M. D., of Edinburgh, and John Harold, M. D., of London Volume IV Tenth series. 1901. Philadelphia: J B Lippincott Company.

A System of Practical Therapeutics. By Eminent American and Foreign Authorities Edited by Hobart Amory Hare, M. D., Professor of Therapeutics, Jefferson Medical College; Physician to Jefferson College Hospital, etc., Philadelphia New (2d) edition, thoroughly revised In three handsome octavo volumes, containing 2593 pages, with 427 engravings and 26 full-page colored plates. Lea Brothers & Co.: Philadelphia and New York 1901. [Price, per volume, cloth, $5.00, net; leather, $6 00, net; half morocco, $7.00, net ]

